# Ghrelin-GHSR-LEAP2 system in the pathophysiology of type 2 diabetes

**DOI:** 10.1016/j.isci.2025.113573

**Published:** 2025-09-15

**Authors:** Yueli Pu, Jianmei Yang, Wei Li, Yi Wen, Chunmei Zheng, Yonglin Li, Lijuan Wu, Yao Ming, Changying Zhao, Chen Chen

**Affiliations:** 1 Endocrinology, The Affiliated Traditional Chinese Medicine Hospital of Southwest Medical University, Luzhou, China; 2 School of Biomedical Sciences, The University of Queensland, Brisbane, Qld 4068, Australia; 3 Department of Pediatric Endocrinology, Shandong Provincial Hospital Affiliated to Shandong First Medical University, Jinan, China; 4 College of Life Sciences, Jilin Agricultural University, Changchun, China

**Keywords:** Endocrinology, Therapeutics, Human metabolism

## Abstract

The Ghrelin-GHSR-LEAP2 system plays a multifaceted role in the pathophysiology of type 2 diabetes mellitus (T2DM). Ghrelin, through the activation of its receptor GHS-R1a, contributes to hyperglycemia by suppressing insulin secretion, inducing insulin resistance, and promoting hepatic glucose production. In contrast, LEAP2, an endogenous antagonist and inverse agonist of GHS-R1a, mitigates these effects by enhancing insulin secretion and improving glucose tolerance. Notably, ghrelin also demonstrates protective properties against diabetic complications through anti-inflammatory, antioxidant, and anti-apoptotic mechanisms. This duality highlights the complexity of the therapeutic targeting of the ghrelin-GHSR axis. This review provides an updated overview of the molecular mechanisms and physiological functions of the ghrelin-GHSR-LEAP2 system in T2DM and discusses the therapeutic potential and challenges of modulating this pathway.

## Graphical abstract

**Figure:**
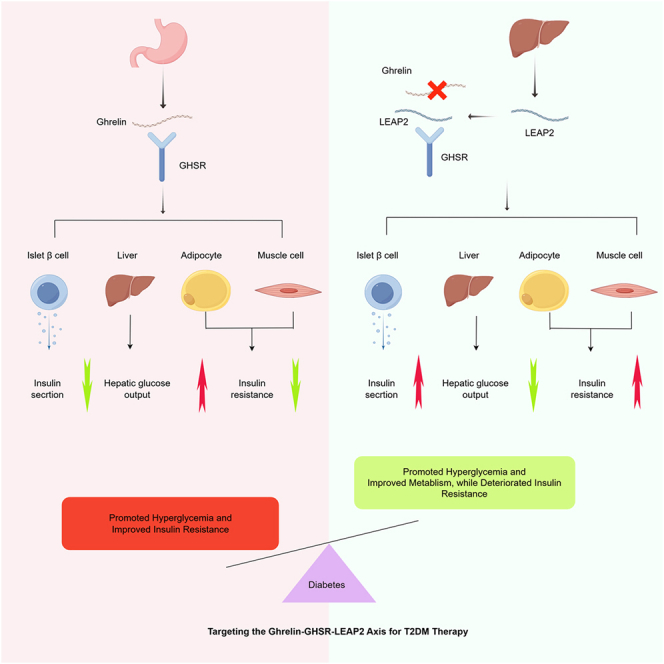

## Introduction

Diabetes mellitus is a metabolic disorder characterized by chronic hyperglycemia. With changing lifestyles and rapid population aging, the prevalence and mortality associated with diabetes are rising sharply. The 10th edition of the Diabetes Atlas reported approximately 537 million adults worldwide living with diabetes in 2021, projected to increase to 643 million by 2030 and 783 million by 2045.^1^ Diabetes poses a significant threat to global health and imposes a heavy societal burden. Type 2 diabetes mellitus (T2DM) accounts for over 90% of all diabetes cases and is characterized primarily by insulin resistance and/or insufficient insulin secretion.

The pathogenesis of T2DM involves a multifaceted interplay of insulin resistance (IR) and progressive β-cells dysfunction in pancreatic islets, often arising within the broader context of metabolic syndrome or metabolic dysfunction syndrome (MDS), which includes conditions such as obesity, dyslipidemia, and nonalcoholic fatty liver disease.^2^ Central to this process is insulin resistance, triggered by dyslipidemia and hyperglycemia, which activate shared pathways such as inflammation, endoplasmic reticulum stress (ERS), oxidative stress, and ectopic lipid deposition, ultimately impairing insulin secretion and signaling.^2^^,^^3^ ERS plays a particularly crucial role in β-cells failure, as disruptions in endoplasmic reticulum homeostasis directly compromise insulin production and contribute to β-cells loss, evident in both monogenic and common forms of diabetes.^2^^,^^4^ Concurrently, oxidative stress and inflammatory pathways, driven by the metabolic dysregulation of glucose and lipids, exacerbate tissue damage and promote complications such as diabetic cardiomyopathy.^3^ Post-translational modifications, including SUMOylation, further modulate the immune microenvironment, influencing inflammatory responses and potentially aggravating T2DM pathogenesis.^5^ Additionally, abnormalities in tryptophan catabolism affect immune and metabolic functions, with disrupted kynurenine and serotonin pathways linked to insulin resistance and vascular complications in diabetes.^6^ Microvascular disease, such as nephropathy or neuropathy, often precedes or accompanies T2DM, reflecting systemic vascular inflammation and endothelial dysfunction that contribute to increased risks of heart failure and other organ damage.^7^ Together, these mechanisms highlight the integrated nature of T2DM pathogenesis, involving genetic, metabolic, and environmental factors that collectively drive disease progression and underscore potential therapeutic targets for intervention.

**Figure 1:**
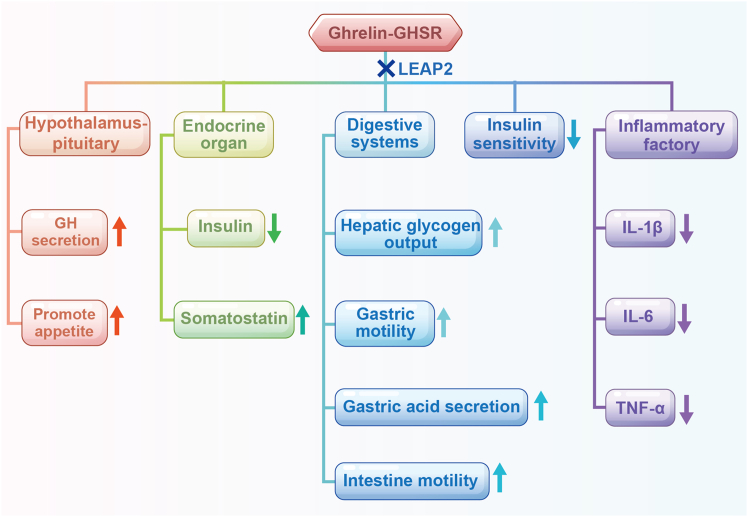
Physiological effects of Ghrelin-GHSR-LEAP2 GHSR: growth hormone secretagogue receptor; GH: growth hormone; LEAP2: liver enriched antimicrobial peptide 2; IL-6: interleukin-6; IL-1β: interleukin-1β; TNF-ɑ: tumor necrosis factor-ɑ. Arrows indicate physiologic roles of ghrelin; Cross indicates that LEAP2 antagonizes or may antagonize these effects.

Ghrelin, initially identified as a GH secretagogue, has been extensively studied in the context of food intake and obesity.^8^^,^^9^ However, ghrelin also plays a vital role in glucose metabolism.^10^ It has been demonstrated to elevate blood glucose levels^11^ and inhibit insulin secretion from pancreatic β-cells.^12^ In peripheral tissues, ghrelin contributes to insulin resistance^13^^,^^14^ and enhances hepatic glycogen output.^15^ The growth hormone secretagogue receptor (GHSR), a G protein-coupled receptor (GPCR), serves as the endogenous receptor for ghrelin. The Ghrelin-GHSR axis participates in diverse physiological functions, including growth hormone (GH) secretion, appetite regulation, and glucose homeostasis (Figure 1).^16^^,^^17^ Consequently, targeting the Ghrelin-GHSR axis has emerged as a potential therapeutic strategy for diabetes and hyperglycemia. For instance, GHSR antagonists have been shown to improve glucose tolerance by enhancing insulin secretion in obesity-related models.^18^ Inhibiting ghrelin via GHSR antagonists reversed diabetes in a mouse model of maturity-onset diabetes of the young 3 (MODY3).^19^ Liver enriched antimicrobial peptide 2 (LEAP2), first isolated in 2003 from human blood ultrafiltrates,^20^ was later identified in 2018 as an endogenous competitive antagonist of the growth hormone secretagogue receptor 1a (GHS-R1a).^21^^,^^22^ LEAP2 may regulate glucose metabolism by the Ghrelin-GHSR axis. Under normal physiological conditions, ghrelin and LEAP2 maintain metabolic equilibrium through reciprocal regulation. However, this balance undergoes pathological disruption in type 2 diabetes^23^ (Figure S1).

## Ghrelin: biosynthesis and active forms

**Figure 2:**
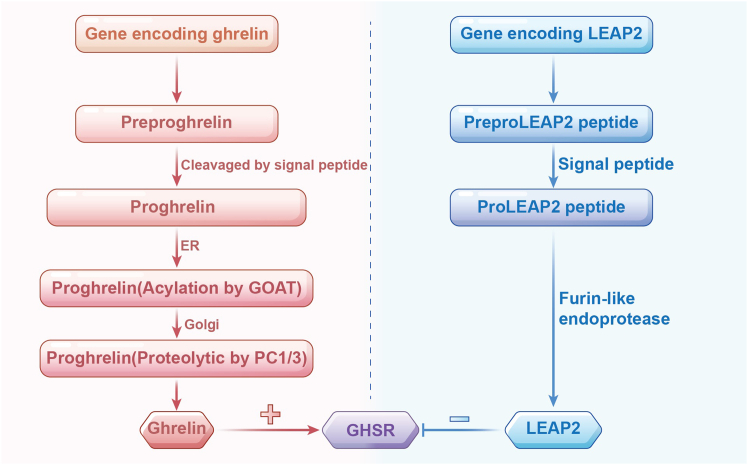
The biosynthesis of Ghrelin and LEAP2 GOAT: ghrelin O-acyltransferase; ER: endoplasmic reticulum; PC: prohormone convertase; LEAP2: liver enriched antimicrobial peptide 2; GHSR: growth hormone secretagogue receptor.

Ghrelin was first characterized by Kojima et al. in 1999 as the endogenous ligand for GHSR.^24^ It is a 28-amino-acid peptide predominantly synthesized and secreted by X/A-like cells in the rodent stomach and P/D1-like cells in humans.^24^ The ghrelin gene encodes a 117-amino-acid preproghrelin peptide, which undergoes cleavage by signal peptidase to generate proghrelin.^24^ Proghrelin is subsequently acylated by ghrelin O-acyltransferase (GOAT) in ER and proghrelin is processed into ghrelin by prohormone convertase (PC) 1/3 in golgi^25^ (Figure 2). Circulating desacyl ghrelin constitutes approximately 90% of total ghrelin, whereas ghrelin is present at much lower concentrations.^26^ The octanoylation of ghrelin is essential for its binding and activation of GHS-R1a,^27^ as GOAT-deficient mice lack circulating ghrelin and fail to activate the receptor.^28^^,^^29^

## GHSR: tissue distribution and signaling mechanisms

GHSR is a G protein-coupled receptor predominantly expressed in the hypothalamus and hippocampus in both humans and rodents.^30^ Beyond the central nervous system, GHS-R1a is expressed in multiple peripheral tissues, including the pituitary, heart, lung, kidney, pancreas, adipose tissue, and intestine.^30^

**Figure 3:**
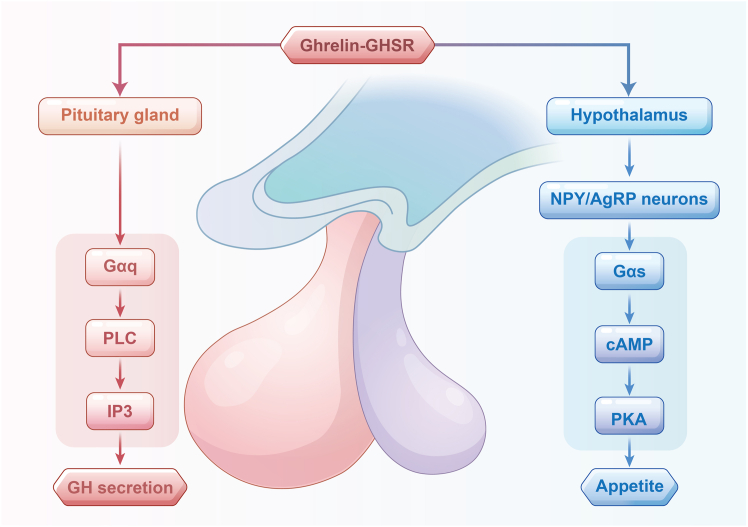
Mechanism of ghrelin promotes appetite and regulates GH secretion NPY/AgRP: neuropeptide Y/agouti-related protein; GH: growth hormone; PLC: phospholipase C; IP3: inositol (1,4,5) triphosphate; cAMP: cyclic adenosine monophosphate; PKA: protein kinase A; GHSR: growth hormone secretagogue receptor.

The GHSR signaling pathway involves both ghrelin-dependent and ligand-independent constitutive activity, mediated through G protein-coupled mechanisms. GHSR is primarily expressed on neuropeptide Y (NPY) and agouti-related peptide (AgRP) neurons in the hypothalamus, where it is activated upon ghrelin binding to promote feeding behavior. Ghrelin binding activates the Gαs-cAMP-PKA signaling cascade, stimulating appetite.^31^^,^^32^ In the pituitary, Ghrelin-GHSR interaction triggers the Gαq-PLC-IP3 pathway, promoting GH secretion (Figure 3).^33^

Constitutive GHSR activity, independent of ghrelin, regulates basal neuronal excitability by modulating voltage-gated calcium channels (e.g., CaV2.2). For instance, GHSR suppresses CaV2.2 currents in the absence of ghrelin, influencing neurotransmitter release and energy homeostasis.^34^ Ghrelin also inhibits pro-opiomelanocortin (POMC) neurons indirectly by enhancing GABAergic synaptic inputs via GHSR-dependent presynaptic mechanisms.^35^

**Figure 4:**
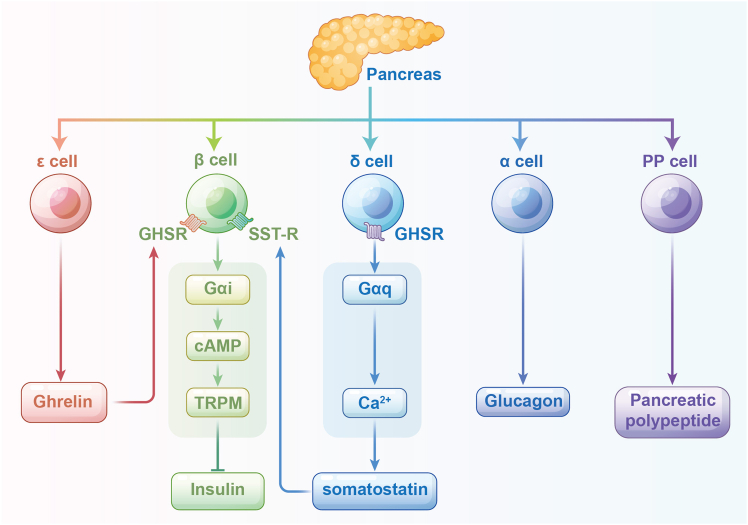
Mechanism of ghrelin inhibits insulin secretion in pancreatic islets SST-R: somatostatin receptor; GHSR: growth hormone secretagogue receptor; cAMP: cyclic adenosine monophosphate; TRPM: transient receptor potential melastatin.

Signaling termination involves β-arrestin recruitment and receptor internalization, processes impaired in GHSR-Q343X mutants, leading to prolonged signaling and metabolic effects such as adiposity.^36^ In pancreatic β-cells, ghrelin suppresses glucose-stimulated insulin secretion through a unique Gαi-cAMP-TRPM2 pathway, reducing intracellular cAMP and blocking calcium influx^37^ (Figure 4). The dysregulation of this pathway contributes to insulin resistance and hyperglycemia in metabolic disorders.

Chronic ghrelin elevation, as seen in Alzheimer’s disease models, induces GHSR desensitization via excessive internalization, linking disrupted signaling to synaptic dysfunction.^38^ Thus, the Ghrelin-GHSR axis integrates nutrient sensing, energy balance, and disease pathophysiology through dynamic, context-dependent signaling mechanisms.

## Liver enriched antimicrobial peptide 2: biosynthesis, structure, and tissue distribution

LEAP2 was first identified in 2003 by Krause et al. as one of the two liver-enriched antimicrobial peptides (LEAP1 and LEAP2) isolated from human blood ultrafiltrates.^20^ While LEAP1 primarily regulates iron metabolism,^39^ LEAP2 has emerged as a key endogenous antagonist of GHS-R1a. The LEAP2 gene in humans comprises three exons and two introns and is located on chromosome 5q31.^20^ The translated LEAP2 precursor is a 77-amino-acid prepropeptide that undergoes proteolytic processing: removal of a 22-residue N-terminal signal peptide yields proLEAP2, which is further cleaved into the mature 40-amino-acid peptide (Figure 2).^40^ The amino acid sequence of LEAP2 is highly conserved across mammalian species.^41^

LEAP2 expression is predominantly localized to the liver and intestine, with the highest levels detected in the jejunum.^21^^,^^42^ Although initially characterized for its antimicrobial properties via interactions with microbial membranes,^40^^,^^43^^,^^44^ the physiological concentrations of LEAP2 (in the nanomolar range) are substantially lower than those required for antimicrobial activity (micromolar range), suggesting its primary role is unrelated to direct antimicrobial defense.

Like ghrelin, LEAP2 secretion is influenced by nutritional status, but in contrast to ghrelin circulating levels increase during fed states or positive energy balance (e.g., obesity) and decrease during fasting or calorie restriction.^41^^,^^45^ The production is also modulated by inflammatory signals and metabolic stressors, reflecting its dual roles in immunity and energy regulation.^46^

The synthesis and secretion of ghrelin and LEAP2 are reciprocally regulated. Ghrelin peaks during fasting to promote hunger, while LEAP2 rises postprandially to antagonize ghrelin signaling. This dynamic balance is reflected in the molar ratio of LEAP2/ghrelin, which shifts according to energy status and plays a critical role in metabolic adaptation. For instance, in obesity, elevated LEAP2 levels suppress ghrelin activity, while in anorexia nervosa, the dysregulation of this ratio disrupts normal appetite control.^41^^,^^47^ These opposing yet interdependent synthesis pathways highlight the importance of the Ghrelin-LEAP2 axis in maintaining energy homeostasis and responding to metabolic challenges.

## Role of the ghrelin-growth hormone secretagogue receptor system in the pathogenesis of type 2 diabetes mellitus

### Ghrelin-growth hormone secretagogue receptor elevates blood glucose and suppresses insulin secretion

Extensive evidence demonstrates that ghrelin raises blood glucose levels in both humans and rodents.^48^^,^^49^ Ghrelin or GHSR knockout mice exhibit reduced blood glucose.^50^ Ghrelin knockout mice require higher glucose infusion, suggesting that ghrelin raises blood glucose.^51^^,^^52^^,^^53^

The hyperglycemic action of ghrelin is largely attributed to its inhibitory effect on insulin secretion.^18^^,^^54^ Elevated serum ghrelin impairs insulin release in mouse models of MODY3, and ghrelin antagonists restore normal glycemic control.^19^ Clinical studies administering exogenous ghrelin to healthy adults reveal suppressed glucose-stimulated insulin secretion (GSIS) alongside elevated blood glucose.^55^^,^^56^^,^^57^ Furthermore, it is found that ghrelin has a direct insulin inhibitory effect in human pancreatic islets.^58^

The pancreas has been reported to consist of five endocrine cell types: glucagon-producing ɑ-cells, insulin-producing β-cells, somatostatin-producing δ-cells, pancreatic polypeptide-producing PP-cells, and ghrelin-producing ε-cells.^59^ In addition to ε-cells, other types of pancreatic endocrine cells also secrete a little of ghrelin.^59^ Single-cell transcriptomic studies in mouse and human pancreatic islets have shown that higher levels of GHS-R1a expression in δ-cells than in β-cells.^60^^,^^61^^,^^62^ Ghrelin activates GHS-R1a on δ-cells and releases somatostatin, which acts in a paracrine manner on β-cells to inhibit insulin secretion.^63^ Studies using *in vitro* cultured β-cells and isolated rat pancreatic islets have shown that ghrelin binds to GHS-R1a of β-cells, signaling downstream via Gαi/o-cAMP-TRPM2 signaling, which in turn enhances delayed K^+^ channels and reduces calcium influx and insulin secretion.^49^^,^^64^^,^^65^ These results revealed that ghrelin from pancreatic islets indirectly/directly inhibits insulin secretion from β-cells in a paracrine and/or autocrine manner (Figure 4).

### Ghrelin-GHSR reduces insulin sensitivity

In addition to suppressing insulin secretion, ghrelin has been shown to reduce insulin sensitivity in peripheral tissues (Figure 2). In ob/ob mice, ghrelin deficiency enhances glucose-stimulated insulin secretion and improves peripheral insulin sensitivity.^54^ Similarly, antagonism of GHS-R1a enhances glucose tolerance in rodent models, potentially through increased glucose-stimulated insulin secretion.^66^ Treatment with GHSR inverse agonists improves glucose metabolism and reduces hyperglycemia in Zucker diabetic fatty (ZDF) rats and diet-induced obese (DIO) mice.^67^^,^^68^

Experimental studies further demonstrate that ghrelin exacerbates hepatic gluconeogenesis and impairs hepatic insulin sensitivity in rats.^69^ Additionally, ghrelin has been shown to affect insulin sensitivity and regulate the counter-regulatory response to insulin-induced hypoglycemia.^70^ Pradhan et al. confirmed that GHSR is expressed in both pancreatic β-cells and δ-cells, where it modulates glucose-stimulated insulin secretion and modulates systemic insulin sensitivity.^71^ In human studies, ghrelin administration induces insulin resistance independently of GH, cortisol, or free fatty acid levels.^13^^,^^72^

Collectively, these findings indicate that the Ghrelin-GHSR system contributes to T2DM pathogenesis not only by suppressing insulin secretion but also by impairing insulin sensitivity. Therefore, the therapeutic inhibition of this pathway may offer metabolic benefits in patients with diabetes by improving both insulin action and secretion.

### The role of growth hormone in glucose and lipid homeostasis

Building on the metabolic actions of the Ghrelin–GHSR axis, its downstream mediator, growth hormone (GH) exerts multifaceted effects on glucose and lipid homeostasis. In the hypothalamus, GH receptor (GHR) signaling in leptin receptor (LepRb)-expressing neurons regulates hepatic insulin sensitivity and lipid metabolism, as mice lacking GHR in LepRb-expressing neurons exhibit the impaired suppression of gluconeogenic genes and disrupted hepatic insulin signaling.^73^ GH also stimulates perinatal β-cells proliferation via Signal Transducer and Activator of Transcription 5 (STAT5)-mediated serotonin production in β-cells, which is critical for establishing adult β-cells mass and maintaining glucose tolerance.^74^ Additionally, GH interacts with testosterone in hypothyroid states to modulate liver transcriptomes involved in lipid biosynthesis and fatty acid metabolism, enhancing GH-regulated pathways linked to unsaturated fatty acid metabolism.^75^ Conversely, ghrelin, a hormone linked to GH secretion, inhibits insulin release and regulates gluconeogenesis, while its receptor (GHS-R) deletion in mice improves thermogenic capacity in brown adipose tissue, suggesting a role in energy expenditure and lipid mobilization.^76^^,^^77^ These findings highlight GH’s integrated roles in glucose regulation, lipid metabolism, and cross-talk with other hormonal systems.

### Protective effects of ghrelin in diabetic complications

The development of diabetic complications is influenced by a complex interplay of genetic, metabolic, hemodynamic, oxidative, inflammatory, and apoptotic factors. In particular, chronic inflammation and oxidative stress form a vicious cycle that drives the progression of diabetic end-organ damage. Ghrelin has demonstrated protective effects in various experimental models by reducing the secretion of proinflammatory cytokines such as interleukin-6 (IL-6), interleukin-1β (IL-1β), and tumor necrosis factor-α (TNF-α), and by inhibiting key inflammatory signaling pathways, including nuclear factor-κB (NF-κB) and NOD-like receptor family pyrin domain containing 3 (NLRP3) inflammasome activation (Figure 2).^78^ These properties highlight its potential to delay or mitigate the onset of diabetic complications.

Ghrelin has shown neuroprotective effects in multiple studies. For example, it reduces neuroinflammation and improves diabetes-associated neurodegenerative changes.^79^^,^^80^ Chronic intracerebral ghrelin infusion in diabetic rats significantly reduced hippocampal neuronal apoptosis, attenuated inflammation, and alleviated streptozotocin-induced cognitive deficits.^81^ *In vitro*, ghrelin inhibits the toll-like receptor 4/nuclear factor-κB (TLR4/NF-κB) signaling pathway, protecting PC12 neuronal cells from high-glucose-induced apoptosis.^82^

In diabetic retinopathy, ghrelin reduces reactive oxygen species (ROS) production, suppresses apoptosis, and inhibits NLRP3 inflammasome activation, thereby protecting retinal cells from high-glucose-induced dysfunction.^83^ In patients with diabetic gastroparesis, relamorelin (a selective GHSR agonist) has been shown to accelerate gastric emptying, reduce vomiting frequency, and improve gastrointestinal symptoms.^84^^,^^85^ In diabetic cardiomyopathy models, ghrelin modulates endoplasmic reticulum stress and inhibits NLRP3 inflammasome activation via the phosphatidylionsitol-3-kinase/protein kinase B (PI3K/AKT) pathway, ultimately preventing cardiomyocyte pyroptosis.^86^

Additionally, elevated ghrelin levels have been observed in patients with chronic kidney disease (CKD), suggesting a role in renal protection. Ghrelin may exert anti-inflammatory effects in CKD, particularly in advanced stages.^87^^,^^88^ Increased ghrelin expression in the distal tubules and collecting ducts of diabetic rats further supports its potential involvement in the pathogenesis of diabetic nephropathy, although the mechanisms remain unclear.^89^

Overall, ghrelin’s beneficial effects on diabetic complications are likely mediated by its anti-inflammatory, antioxidant, and anti-apoptotic properties. However, most of the current evidence is derived from animal studies; clinical trials are lacking. Therefore, further research is needed to elucidate the precise molecular mechanisms and validate these findings in human subjects.

### The inconsistencies in ghrelin’s role in glucose metabolism

The inconsistencies in ghrelin’s role in glucose metabolism stem from its complex dual actions in central and peripheral pathways, as well as variations between fasting and postprandial states, necessitating the clearer classification of research to resolve ambiguities. Ghrelin primarily suppresses insulin secretion from pancreatic β-cells and increases hepatic glucose output, promoting hyperglycemia in various contexts. For instance, studies show that the ghrelin inhibition of insulin release involves AMPK-UCP2, ATP-sensitive potassium channels, and altered intracellular calcium levels, which may impair glucose tolerance, particularly in obesity, to exacerbate diabetic phenotypes.^90^^,^^91^ However, this hyperglycemic effect is inconsistent with findings where ghrelin receptor knockout (KO) or neuronal deletion of GHS-R (ghrelin receptor) significantly improves insulin sensitivity, enhances glucose disposal, and reduces hepatic glucose production, suggesting that central ghrelin signaling primarily drives metabolic dysfunction, such as diet-induced obesity and insulin resistance, while ablation models reveal protective glucose-lowering outcomes.^92^^,^^93^ These discrepancies highlight the need to delineate central versus peripheral mechanisms; central ghrelin, acting through hypothalamic neurons (e.g., GHS-R in NPY/AgRP neurons), influences appetite and growth hormone secretion, indirectly disrupting glucose homeostasis and contributing to insulin resistance during metabolic stress,^90^^,^^92^ whereas peripheral ghrelin, produced by the stomach or pancreas, directly targets β-cells to inhibit insulin secretion and to promote gluconeogenesis in the liver, as evidenced by elevated malondialdehyde and impaired insulin responses in diabetic models.^90^^,^^91^^,^^94^

Moreover, the metabolic effects of ghrelin vary markedly between fasting and postprandial conditions, further complicating interpretations. In fasting states, ghrelin levels rise to stimulate feeding and mobilize energy reserves, increasing hepatic glucose production to prevent hypoglycemia, which serves as a critical survival mechanism but may lead to hyperglycemia if improperly elevated, as shown in calorie-restricted or insulin-deficient scenarios.^90^^,^^91^ In contrast, postprandial ghrelin suppression is essential for optimal glucose disposal and insulin sensitivity; yet, persistent improper post-meal ghrelin activity (e.g., in obesity) continues to inhibit insulin action and reduce peripheral glucose uptake in tissues such as muscle and adipose tissues, impairing glucose tolerance. Experimental interventions, such as ghrelin administration in diabetic rats, demonstrate anti-apoptotic and antioxidant effects that improve pancreatic function and glucose homeostasis, suggesting food-intake-dependent protective roles that differ from the fasting actions of ghrelin.^91^^,^^93^^,^^94^ To reconcile these inconsistencies, future research must explicitly categorize studies by site of action (central vs. peripheral) and metabolic state (fasting vs. postprandial), as these differences may elucidate details underlying the dual roles of ghrelin on glucose metabolism and identify targeted therapies for obesity-related insulin resistance.^90^^,^^92^^,^^95^

Interestingly, while the Ghrelin-GHSR axis may exacerbate hyperglycemia and insulin resistance, it simultaneously confers protection against long-term diabetic complications. This duality underscores the need for a nuanced understanding and targeted modulation of the pathway in therapeutic development.

## Liver enriched antimicrobial peptide 2 as an inverse agonist and competitive antagonist of growth hormone secretagogue receptor 1a

The Ghrelin-GHSR axis is involved in multiple physiological processes, including appetite regulation, GH secretion, and glucose homeostasis. LEAP2, identified as an endogenous antagonist of GHS-R1a, has garnered significant attention for its potential to modulate these processes, particularly in the context of metabolic disorders such as type 2 diabetes.

### Mechanisms of liver enriched antimicrobial peptide 2 action on growth hormone secretagogue receptor 1a

In 2018, Ge et al. first reported that LEAP2 functions as a noncompetitive antagonist of GHS-R1a, effectively inhibiting ghrelin-induced GH secretion.^21^ Later, Wang et al. clarified that LEAP2 competes directly with ghrelin for binding to GHS-R1a, suggesting a shared ligand-binding site.^22^ Interestingly, LEAP2 exhibits time-dependent antagonist behavior: when applied simultaneously with ghrelin, it acts competitively; when pre-incubated, it behaves as a noncompetitive antagonist—likely due to its slow dissociation rate from the receptor. Further supporting this dual mechanism, Barrile et al. developed a fluorescent LEAP2 analog (F-LEAP2), which displayed similar binding affinity and inverse agonist activity toward GHS-R1a *in vitro*.^96^ LEAP2 has been shown to counteract ghrelin’s effects on appetite, GH release, fasting glucose maintenance, and insulin suppression (Figure 2).^21^^,^^41^^,^^42^

### Liver enriched antimicrobial peptide 2 counteracts ghrelin-induced insulin suppression

In isolated rat islets, LEAP2 effectively antagonizes the ghrelin-induced suppression of insulin secretion.^97^ *In vivo*, LEAP2 administration reduces blood glucose levels in wild-type mice, but not in GHSR-knockout mice, indicating a receptor-dependent mechanism.^98^ Notably, the N-terminal fragment of LEAP2 (LEAP2_38-47_) also demonstrated its potent pro-insulinotropic effects *in vitro.*^99^^,^^100^ Recent studies suggest that LEAP2 and ghrelin exert opposing and sex-dependent effects on insulin secretion. In male mice, this interaction is modulated by estradiol.^101^ In diabetic mouse models, LEAP2 administration improves glucose tolerance, potentially by enhancing glucose-stimulated insulin secretion through the GHSR-PPARγ (peroxisome proliferator-activated receptor γ) signaling pathway.^102^

Clinical results also support LEAP2’s role in glucose metabolism. Healthy men received exogenous injections of LEAP2, which was shown to reduce postprandial blood glucose fluctuations and inhibit food intake. Experiments with GHSR knockout mice suggest that these effects may be mediated by GHSR.^98^ Cross-sectional studies report increased serum LEAP2 levels in individuals with type 2 diabetes, which positively correlate with glycated hemoglobin (HbA1c) levels.^103^ Moreover, LEAP2 has been associated with improved insulin secretory function in overweight and obese individuals.^104^ These findings highlight the potential of LEAP2 as a novel therapeutic agent for diabetes.

However, LEAP2 may act through additional receptors. For example, in calorie-restricted GHSR-knockout mice, chronic LEAP2 treatment still induced weight loss and hypoglycemia,^105^ implying the existence of GHSR-independent pathways. Further investigation is needed to fully characterize LEAP2’s mechanism of action and off-target effects.

### Anti-inflammatory actions of liver enriched antimicrobial peptide 2 in metabolic regulation

LEAP2 exhibits dual roles in modulating inflammation and glucose metabolism, with potential implications for diabetes pathogenesis.^104^^,^^106^ For patients with prediabetes or poorly controlled type 2 diabetes and obesity, plasma LEAP2 levels are positively correlated with body mass index, plasma insulin, blood glucose, and glycated hemoglobin, while negatively correlated with insulin resistance.^41^^,^^107^ In adults with obesity, elevated LEAP2 levels correlate with reduced NF-κB activity in peripheral blood mononuclear cells, suggesting anti-inflammatory properties through the suppression of pro-inflammatory NF-κB signaling.^104^ This interaction may mitigate chronic low-grade inflammation, a key driver of insulin resistance in type 2 diabetes. Paradoxically, LEAP2 is also positively associated with insulin secretion in obesity,^104^ potentially compensating for insulin resistance by enhancing pancreatic β-cells function. While no direct association of LEAP2 with fasting glucose, the dual modulation of LEAP2 on the inflammatory signal pathway and the insulin secretion process also serves as a molecular bridge between metabolic inflammation and abnormal glycemia, warranting further investigation into its therapeutic potential for diabetes management.

## Conclusions

**Figure 5:**
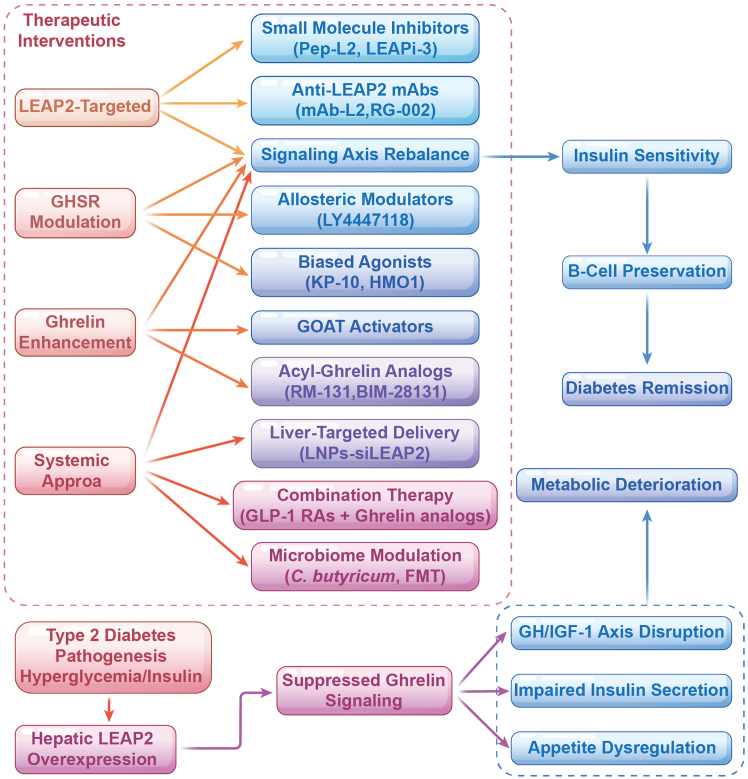
Flowcharts illustrating proposed intervention strategies targeting this axis in T2DM GHSR: growth hormone secretagogue receptor; GH: growth hormone; LEAP2: liver enriched antimicrobial peptide 2; IGF-1: insulin-like Growth Factor-1; GOAT: ghrelin O-acyltransferase; FMT: Fecal Microbial Transplantation; GLP-1 RA: glucagon-like peptide-1 receptor agonist.

The Ghrelin-GHSR-LEAP2 system represents a complex and dynamic regulatory network in the pathophysiology of T2DM (Figure 5). Ghrelin exacerbates hyperglycemia by inhibiting insulin secretion and promoting insulin resistance, while paradoxically exerting protective effects against diabetic complications through its anti-inflammatory, antioxidant, and anti-apoptotic properties. This contradictory nature of ghrelin raises critical questions about its precise role in diabetes progression and complications, which necessitate further *in vivo* studies in diabetic complication models to determine whether ghrelin’s protective actions outweigh its deleterious metabolic effects.

Conversely, LEAP2 functions as an endogenous antagonist and inverse agonist of GHS-R1a, counterbalancing ghrelin’s hyperglycemic actions and demonstrating therapeutic potential in improving glucose tolerance and β-cells function. However, there are still no clinical studies investigating the use of LEAP2 in patients with diabetes. Future research should include extensive *in vivo* experiments in diabetes models to validate LEAP2’s efficacy and safety, laying the groundwork for potential clinical trials to establish LEAP2 as a therapeutic agent for T2DM.

Moreover, it remains unknown whether LEAP2’s appetite-suppressing effects could synergize with existing incretin-based therapies such as GLP-1 receptor agonists, or whether they might lead to overlapping side effects. Exploring these potential interactions should be a research priority.

Targeting this system, particularly through LEAP2-based or combined ghrelin/LEAP2 modulation, holds promise for developing innovative treatments for T2DM. Nevertheless, several barriers to clinical translation persist, including the need to reconcile ghrelin’s dual actions, optimize drug design for receptor selectivity and stability, and avoid systemic side effects. Future research should prioritize elucidating the tissue-specific and context-dependent roles of both ghrelin and LEAP2, advancing pharmacological development, investigating potential combination therapies with existing antidiabetic agents, and validating these findings through well-designed large-scale human clinical trials. As a multifactorial modulator of glucose homeostasis and diabetic complications, the Ghrelin-GHSR-LEAP2 system warrants deeper investigation as a novel therapeutic target for type 2 diabetes.

### Limitations of the study

Although this review provides a systematic overview of the Ghrelin–GHSR– LEAP2 axis in the context of type 2 diabetes, several conceptual and methodological limitations should be noted. Much of the current understanding originated from animal studies and *in vitro* models, which did not comprehensively mirror human metabolic physiology or the multifactorial etiology of human diabetes. Furthermore, robust interventional data in humans, particularly regarding the therapeutic efficacy and safety of ghrelin and LEAP2, are still missing. The pleiotropic and often antagonistic actions of ghrelin on glycemic control and complication-related pathways pose substantial challenges for pharmacological development. In addition, the potential existence of non-canonical, GHSR-independent signaling mechanisms for LEAP2 has not been exhaustively investigated and may present unforeseen translational obstacles. Finally, gender dimorphism in signaling responses and the long-term consequences of the systemic modulation of this endocrine axis remains poorly defined and merits focused examination in future studies.

## Acknowledgments

Work has been funded by the NHMRC and Qld-CAS collaborative grant (to CC), the 10.13039/501100007129Natural Science Foundation of Shandong Province (CN) (ZR2024MH184) (to JMY), the Luzhou Science and Technology development grant (2024WGR205), The Affiliated Traditional Chinese Medicine Hospital of 10.13039/501100014895Southwest Medical University, China; NHMRC, Australia; and the 10.13039/501100001794University of Queensland, Australia; the 10.13039/501100001809National Natural Science Foundation of China (82100920) (to JMY); and the Young and Middle-aged Innovative Talents Project of Shandong Provincial Hospital (to JMY). The images in this article were drawn by Figdraw software.

## Author contributions

C.C. conceived and designed the study. Y.L.P., J.M.Y., Y.W., C.M.Z., Y.L.L., L.J.W., Y.M., and C.Y.Z. reviewed the literature and participated in the drafting and writing of the article. J.M.Y. and Y.L.P. created figures for the article. C.C., J.M.Y., and Y.L.P. edited the final version of the article. All authors read and approved the final version of the article and agree with the order of presentation of the authors.

## Declaration of interests

Authors declare no competing interests.
