# Supplementary material for: Ghrelin-GHSR-LEAP2 system in the pathophysiology of type 2 diabetes
Source: iScience. 2025 Sep 15;28(10):113573. doi: 10.1016/j.isci.2025.113573 (PMC12506571; doi:10.1016/j.isci.2025.113573)

iScience, Volume *28*

Supplemental information

Ghrelin-GHSR-LEAP2 system

in the pathophysiology of type 2 diabetes

Yueli Pu, Jianmei Yang, Wei Li, Yi Wen, Chunmei Zheng, Yonglin Li, Lijuan Wu, Yao Ming, Changying Zhao, and Chen Chen

Supplement Figure 1. Schematic diagram about balance of Ghrelin and LEAP2. This review summarizes current understanding of the Ghrelin-GHSR-LEAP2 system in glucose metabolism and explores its therapeutic potential in T2DM.

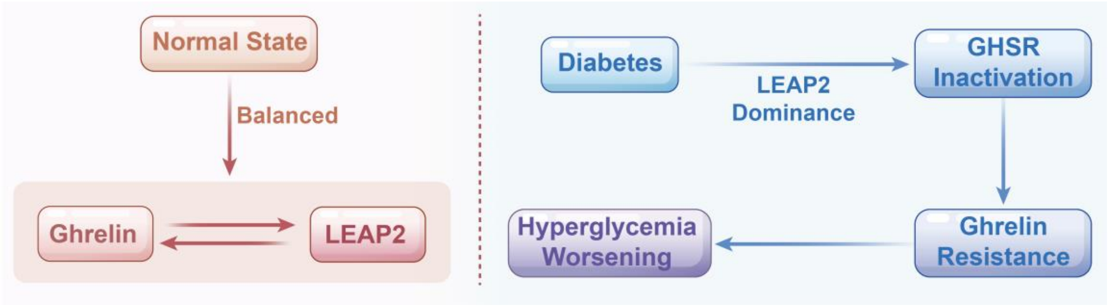

Supplement: Document S1. Figure S1 [file mmc1.pdf]
